# Effect of *Zataria multiflora* Boiss. essential oil, NaCl, acid, time, and temperature on the growth of *Listeria monocytogenes* strains in broth and minced rainbow trout

**DOI:** 10.1002/fsn3.2208

**Published:** 2021-02-27

**Authors:** Setayesh Hosseini, Esmail Abdollahzadeh, Vahid Ranaei, Maryam Mahmoudzadeh, Zahra Pilevar

**Affiliations:** ^1^ Department of Cell and Molecular Biology Sciences School of Biology College of Science University of Tehran Tehran Iran; ^2^ International Sturgeon Research Institute Agricultural Research, Education and Extension Organization (AREEO) Rasht Iran; ^3^ Social Determinants in Health Promotion research Center Hormozgan Health Institute Hormozgan University of Medical Sciences Bandar Abbas Iran; ^4^ Nutrition Research Center and Department of Food Science and Technology Faculty of Nutrition and Food Science Tabriz University of Medical Sciences Tabriz Iran; ^5^ Department of Food Sciences & Technology Faculty of Nutrition Sciences and Food Technology National Nutrition & Food Technology Research Institute Shahid Beheshti University of Medical Sciences Tehran Iran

**Keywords:** acidification, Bioscreen, growth, *Listeria monocytogenes*, NaCl, rainbow trout, zataria multiflora essential oil

## Abstract

The small outbreaks of listeriosis as one of the leading causes of food poisoning‐associated deaths occur more than previously reported. In current study, the growth ability of *Listeria monocytogenes* strains isolated from different sources of food and human origin was measured under salt stress (0.5%, 2.5%, 5%, 7.5%, and 10%) and acid environments (pH = 6.64 and 5.77) for 96 hr by using a Bioscreen C microbiology reader at 37°C. In further steps of this study, after analysis of constituents of *Zataria multiflora* Boiss. essential oil (ZMEO), the sensory evaluation of the treated fish meat with ZMEO was performed. Then, the fish isolate of *L. monocytogenes* was exposed to sensory acceptable and subminimum inhibitory concentrations (subMICs) of ZMEO in fish broth and minced fish meat during incubation at abuse (12°C), room (22°C), and optimum (37°C) temperatures for 48 hr. The MIC of NaCl against four strains of *L. monocytogenes* was 10% at 37°C. The maximum optical densities (ODs) and under curve areas (AUC) of growth patterns in higher pH value and lower contents of NaCl followed the order of 21C > 6F > 66C > 22C of *L. monocytogenes* strains, while the lag time was prolonged in the reverse order. The maximum OD, growth, and lag times of samples treated with higher contents of NaCl and lower pH value were affected in a different order. The organoleptic evaluation showed that the fish meat treated with less than 0.5% of ZMEO was sensory acceptable. The population of *L. monocytogenes* remained relatively constant at the inoculation level of 10^7^ cfu/ml (or g) at 12°C in broth and minced fish mediums. The inhibitory antilisterial activity of essential oil as an extensive‐used plant for food and pharmacological applications is negligible due to possible adverse sensory and toxic effects at relevant high doses.

## Graphical abstract

*Listeria monocytogenes* as the causative agent of listeriosis was exposed to subinhibitory concentrations (subMICs) of Zataria multiflora Boiss. essential oil (ZEO) in fish broth and minced fish during incubation at abuse (12°C), room (22°C), and optimum (37°C) temperatures for 48 hr. The increase in temperature significantly decreased the antimicrobial efficiency of ZEO.

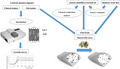

## 1 INTRODUCTION

*Listeria monocytogenes* is an ubiquitous psychrotrophic Gram‐positive bacteria entering food by cross‐contamination during preparation or processing through raw materials and environment like improperly sanitized equipment or workers with poor‐hygiene. The growth of *L. monocytogenes* is influenced by several intrinsic and extrinsic factors in vivo, such as temperature and pressure fluctuations, water activity (a_w_), moisture and oxygen contents, interaction with food components, and presence of competitive micro flora (Abdollahzadeh et al., 2016). This bacteria causes two severe invasive infections of maternal–neonatal and central nervous system with high rates of hospitalization and mortality (20%–30%) (Abdollahzadeh et al., 2014). These infections can be a serious hazard for susceptible populations or YOPI (young, old, pregnant, immune‐compromised individuals) (Lomonaco et al., 2009). Almost all the listeriosis cases are known to be foodborne, which remarkably originate from ready‐to‐eat (RTE) foods, mainly those without further heat treatments such as fish products (Ricci et al., 2018). Fish products can be contaminated at any point in the farm to fork value chain. Abdollahzadeh et al. found a *L. monocytogenes* contamination rate of 8.86% for Iranian fish products (Abdollahzadeh et al., 2016).

*Zataria multiflora* Boiss. with the local name of Shirazi thyme is a member of Lamiaceae family. Several studies have indicated the antiseptic, antioxidative, antibacterial, and therapeutic role of *Z. multiflora* essential oil (ZMEO). Similar to *Thymus vulgaris* essential oil, ZMEO consists of high contents of oxygenated monoterpenes such as thymol and carvacrol. These two components are able to disintegrate the outer membrane of bacterial cells by different mechanisms of action. Despite the vast application of ZMEO in food industry, rare research has been carried out on the use of ZMEO at acceptable sensory levels as the antilisterial flavoring agent in real food. In the recent years, there have been a great tendency to reduce application of sodium chloride in foods due to its role in hypertension. On the other hand, application of NaCl as a distinctive cheap additive is an aspect of osmotic hurdle to reduce water activity in foods. In the current study, the effect of salt and acid on the growth of food and clinical *L. monocytogenes* strains was compared upon exposure to NaCl concentrations of 0.5%, 2.5%, 5%, 7.5%, and 10% and acid environments (pH = 6.64 and 5.77) for 96 hr by using a Bioscreen C microbiology reader at 37°C (step 1). Previous studies have shown extensive variations in resistance of *L. monocytogenes* strains under stress conditions (Lis‐Balchin & Deans, 1997). After comparing the food and clinical strains of *L. monocytogenes* under acid and salt stress by using Bioscreen C microbiology reader, composition, and sensory analysis of *Zataria multiflora* Boiss. essential oil (ZMEO) as a common essential oil in meat industry was obtained (step 2). Afterward, the *L. monocytogenes* strain isolated from rainbow trout fish was exposed to ZMEO in rainbow trout fish broth and minced rainbow trout fish meat as real foods (step 3). There are few studies investigating the growth of fish *L. monocytogenes* isolates in broth and minced fish mediums. In current study, the growth of *L. monocytogenes* was studied at abuse, room, and optimum temperatures in fish broth medium and minced fish medium containing sensory acceptable and sublethal concentrations of ZMEO for 48 hr. This comparison provides information regarding the influence of the time and growth medium (i.e., fish broth and minced fish) on the growth of *L. monocytogenes* isolated from fish when exposed to ZMEO during incubation time (48 hr) at three temperatures (12, 22, and 37°C).

## 2 MATERIALS AND METHODS

### 2.1 Bacteria

The *L. monocytogenes* strain isolated from rainbow trout samples (6F) was used throughout this study. The polymerase chain reaction assays and *inlA* and *prs* primers were employed to detect *L. monocytogenes* isolates from fish (data not shown), and the clinical *L. monocytogenes* strains (22C, 66C, and 21C) were obtained from the Microbiology Department of the Iran University of Medical Sciences, Tehran, Iran.

### 2.2 Behavior of *Listeria monocytogenes* at various NaCl concentrations and pH values

The growth ability of *L. monocytogenes* upon exposure to NaCl concentrations of 0.5%, 2.5%, 5%, 7.5%, and 10% and by acidification (pH: 6.64 and 5.77) was studied via measuring the optical densities at 600 nm (OD_600_) by using a Bioscreen C microbiology reader (Oy Growth Curves Abt Ltd, Helsinki, Finland) at 37°C for 96 hr. The isolates of *L. monocytogenes* were inoculated at 37°C for 24 hr in BHI broth (Merck, Germany), for all the experiments in this study. The cells were harvested by three times centrifugation at 1,000 *g* for 5 min. The re‐suspended cells in physiological serum were adjusted to 10^8^ cfu/ml by reaching OD_600nm_ of 0.08–0.1 (UV spectrophotometer, Cecil, UK). Aliquots of 400 µl of bacterial dilution (3.28 log cfu/mL) at each NaCl concentrations (0.5%, 2.5%, 5%, 7.5% and 10%) and pH values (5.77 and 6.64) were placed in the wells of 96‐well honeycomb plates. Then, the plates were incubated in the Bioscreen C microbiology reader. The linear shaking was provided for every 15 min to have a homogenous mixture before each measurement. All experiments were done in duplicates.

### 2.3 Fish meat

The rainbow trout (*Oncorhynchus mykiss*) fish was purchased from a local supermarket and was transported to the laboratory in ice boxes. For homogeneity of fish samples, the skin was aseptically removed and muscles were ground by a blender in sterile conditions. The fish was wrapped and kept at refrigeration temperature. The frozen‐thawed samples were used for microbiological assays.

### 2.4 Fish broth

To prepare fish broth, the fish samples were boiled in the ratio of 1:2 (w/v) with distilled water for 100 min. Then, the suspension was filtered and buffered by 10 g/L of K_2_HPO_4_ and 6 g/L of KH_2_PO_4_. Before use, the broth was sterilized at 121°C for 15 min.

### 2.5 Essential oil

The commercial essential oil of *Zataria multiflora* Boiss. was used throughout this study. This oil was purchased from Gol‐ghatreh Co.

### 2.6 GC‐MS

The diluted ZMEO (1:5 in ethanol, v/v) was injected in a split‐split less mode. Then, the analysis of chemical components of ZMEO was run on a gas chromatography–mass spectrometry (GC‐7890, MS‐5975) systems of Agilent Technologies, Santa Clara, CA, USA. Helium with a constant rate of 0.5 ml/min was used as the carrier gas. The HP‐5 capillary column (30 m × 250 μm × 0.25 μm) was directly coupled to the MS apparatus. The initial temperature of column was programmed for 50°C, and then gradually was increased to 180°C at a 5°C/min rate and finally increased to 310°C at 10°C/min. The identification of constituents was done by comparing mass spectra using NIST software. The relative proportion of components or the peak area of components relative to total peak area of all components was expressed as percentage.

### 2.7 Sensory evaluation

The sensory evaluation of the treated minced fish samples was done by an experienced 9‐member panel in a sensory laboratory at room temperature. The panelists who were familiar with fish products were recruited among the faculty members of Shahid Beheshti University of Tehran, Iran. The portions of minced fish samples (200 g) were treated with different concentrations of ZMEO (0.3%, 0.5%, and 0.7%) and were labeled with three digits. The fish samples in aluminum foils were steamed in a steam cooker at 100°C for 15 min and were served on white plates. The panelists were asked to rate how they liked/disliked the sample overall acceptance, flavor, color, odor, and texture. The panelists were instructed to cleanse their palate by using water and low‐salt crackers prior to the testing and between the assessments. The five‐point hedonic scale from 1 to 5, where 1 = dislike extremely, 2 = dislike moderately, 3 = neither like nor dislike, 4 = like moderately, and 5 = like extremely was used.

### 2.8 Growth upon exposure to *Zataria multiflora* Boiss. essential oil

To assess the effect of ZMEO on the growth of *L. monocytogenes*, the concentrations of ZMEO were selected on the basis of our previous study. As results of previous study showed, the MICs of ZMEO against *L. monocytogenes* incubated at three temperatures of 12°C, 22°C, and 37°C were 0.031%, 0.0625%, and 0.09%, respectively. The MIC values obtained in minced fish meat were 0.8%, 1.4%, and 2.1%, respectively (Pilevar et al., 2020). In the current study, after addition of selected concentrations of ZMEO (subMICs), which were 0.01 and 0.5% for fish broth and minced fish meat, respectively, incubation of samples was done for 48 hr at three temperatures of 12°C, 22°C, and 37°C. To dissolve ZMEO in fish broth, 0.15% of agar was used. For final inoculum of 10^7^ cfu/ml, different volume of 10^8^ cfu/ml (OD: 0.08–0.1) bacterial suspension was added to solutions containing different concentration of ZMEO.

For serial dilutions, 5 g of fish samples were homogenized with 45 ml of physiological serum. The population of *L. monocytogenes* was enumerated by pour plating 1 ml of selected dilutions in PALCAM Listeria Selective medium (Merck, Germany). Then, the plates were incubated at 37°C for 24 hr. The positive and negative controls, to confirm the viability of bacterial cells and check the sterility, respectively, were prepared. Each experiment was carried out in duplicate.

### 2.9 Statistical analysis

The data exploration and plotting of Bioscreen C microbiology reader was obtained by using Microsoft Excel 2013 (Microsoft Corporation). For comparison of sensory analysis, Kruskal–Wallis was performed between the groups by using SPSS software v. 22 (SPSS Inc.). *p*‐values lower than .05 were considered as statistically significant.

## 3 RESULTS AND DISCUSSION

**FIGURE 1:**
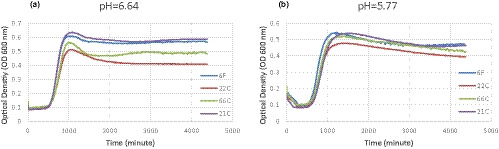
Growth ability of *Listeria monocytogenes* strains (6F, 22C, 66C and 21C) upon exposure to acidification (A: 6.64, B: 5.77)

**FIGURE 2:**
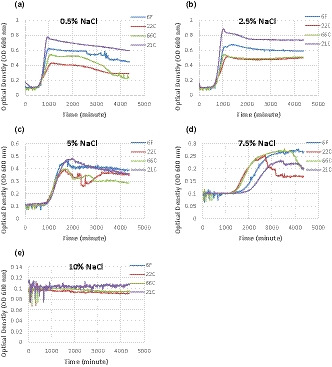
Growth ability of *Listeria monocytogenes* strains (6F, 22C, 66C and 21C) upon exposure to NaCl concentrations of 0.5% (a), 2.5% (b), 5% (c), 7.5% (d), and 10% (e)

Figures 1 and 2 show the acid and salt tolerance of *L. monocytogenes* upon exposure to different concentrations of HCl (%) (pH values of 6.64 and 5.77) and NaCl (0.5%, 2.5%, 5%, 7.5%, and 10%) at 37°C. As shown in Figures 1 and 2, both maximum optical densities and under curve areas (AUC) followed the order of 21C > 6F > 66C > 22C of *L. monocytogenes* strains, while the lag time was prolonged in the reverse order. The maximum OD, growth, and lag times of samples treated with higher contents of NaCl (7.5%) and higher contents of acid (pH: 5.77) were affected in a different order. Variation of growth patterns between *L. monocytogenes* strains was more apparent at 7.5% NaCl and at pH: 5.77 than lower contents of salt and acid. The higher the NaCl and acid concentration, the more extended was the length of the lag time. In Vialette et al. study, it was shown that there were no differences between fish and clinical strains of *L. monocytogenes* upon exposure to acid or salt at 20°C (Vialette et al., 2003). In accordance with the study of Ref. (Aalto‐Araneda et al., 2020), the susceptible strains showed lower growth rates and maximum OD and longer lag times than tolerant strains. *Listeria monocytogenes* can survive or even grow under severe conditions such as high NaCl concentrations up to 10% or in a wide pH range of 4.7–9.2 (Magalhães et al., 2016). The closely genetic related strains of *L. monocytogenes* exhibit similar phenotypes under stress conditions (Bergholz et al., 2010). As reported, there are overlaps between regulatory factors of *L. monocytogenes* in stress response. Therefore, exposure to a stress factor can lead to cross‐protective effect against other stress factors (Tiganitas et al., 2009). Given that susceptibility of *L. monocytogenes* strains can be quantified by growth parameters derived by modeling bacterial growth (Abdollahzadeh et al., 2017; Peleg & Corradini, 2011), relative comparison of strains via growth patterns can be done by measuring and comparing the optical densities using Bioscreen C reader (Pla et al., 2015). However, it should be noted that changes in cell size and morphological changes can occur during growth under high stress conditions (e.g., presence of salts or antibiotics) (Bereksi et al., 2002). The increase in cell size can increase the OD even though there is no increase in cell numbers (Stevenson et al., 2016). However, under NaCl stress and acidification the filaments formed by elongation of *L. monocytogenes* are composed of several normal size cells on the verge of division by septa (Bereksi et al., 2002; Hazeleger et al., 2006). As reported, NaCl concentrations of more than 5.5% cause the production of filaments with multiple septa in *L. monocytogenes* (Geng et al., 2003). Therefore, the increase of OD under salt stress can be caused by such filaments which increase *L. monocytogenes* cell numbers. In favor conditions, the filaments can split into many cells which can contaminate foods (Hazeleger et al., 2006).

*Listeria monocytogenes* can persist severe stress conditions in food and cause foodborne listeriosis. As reported, *L. monocytogenes* can employ adaptive mechanisms under severe stress and grow at pH of 1–4 and higher concentrations of salt (>10%) (Chan & Wiedmann, 2008; Cornu et al., 2006; Shabala et al., 2008; Shah & Bergholz, 2020). In this study, NaCl concentration of 10% inhibited the growth of all food and clinical strains of *L. monocytogenes* (Figures 1 and 2). Therefore, the minimum inhibitory concentration of NaCl against *L. monocytogenes* is 10% at 37°C. Given that *L. monocytogenes* is relatively resistant to saline conditions, however, the overall suboptimal conditions of foods including pH, temperature, and disinfectants may alter the growth patterns in NaCl osmotic stress (Magalhães et al., 2016; Veen et al., 2008). For example, lower temperatures can be associated with decrease in resistance of *L. monocytogenes* to sodium chloride (Faleiro et al., 2003). In contrast, acid adaption can induce cross‐protection and increase the osmotolerance response in *L. monocytogenes* (Faleiro et al., 2003). Prior exposure to osmotic stress can cause cross‐protection against low pH. This adaption to subsequent stress conditions can be associated to *sigB*‐ and SigB‐regulated genes in *L. monocytogenes* (Begley et al., 2002, 2005; Bergholz et al., 2012b; Zhang et al., 2011). However, the result of current study showed that adaption to osmotic and acid tolerances of *L. monocytogenes* can be different among different strains. The stress tolerance strains of *L. monocytogenes* can be identified by relative comparison of growth curves between strains. The lag and growth phases were prolonged by supplementation of NaCl as well as acidification. In accordance to the result of (Bereksi et al., 2002), the lag time of *L. monocytogenes* was increased with acidification of growth medium. The tolerant strains of *L.monocytogenes* adapt more rapidly to severe conditions including moderate acid and salt concentrations (Magalhães et al., 2016). However, the persistent strain of *L.monocytogenes* isolated from fish slaughter was more susceptible to stressors during food processing than those clinical or reference strains (Porsby et al., 2008). Ringus et al. concluded that there is no correlation between tolerance and transcription levels of regulatory genes such as σ^B^ and CtsR in *L.monocytogenes* strains isolated from fish (Ringus et al., 2012).

In second step of this study, ZMEO was analyzed. The main constituents of ZMEO which compromised 97.8% of oil were from phenolic monoterpenes of carvacrol (30.50%) and thymol (29.1%), followed by p‐cymene (11.45%) and γ‐terpinene (9.30%). The minor components were β‐caryophellene (sesquiterpene, 5.80%), α‐pinene (4.08%), α‐terpinene (3.79%), camphor (2.82%), β‐pinene (2.16%), and linalool (1.0%). Various factors affect EOs constituents such as species and part of plant, geographical, and seasonal variations, edaphic changes, plant's production and storage conditions, extraction methods, injuries, water stress, and droughts. (Pilevar & Hosseini, 2013; Pilevar et al., 2017). However, in nearly all of studies, the major constituents of ZMEO are of oxygenated monoterpenes including carvacrol, thymol, and p‐cymene (Kashiri et al., 2017; Moradi et al., 2016). Other than volatile compounds, the nonvolatile compounds (e.g., flavonoids) with anti‐inflammatory role might also exhibit antimicrobial properties (Sajed et al., 2013).

In accordance to the results of current study, (Abdollahzadeh et al., 2014) have reported high concentrations of EO needed to suppress the growth of *L. monocytogenes*. The Iranian standards and USDA legislations have imposed a zero‐tolerance policy for *L. monocytogenes* in RTE food products such as fish products. The interactions of fish components such as protein and lipids with phenolic compounds of carvacrol and thymol results to reduction in their antimicrobial efficiency. This might be the important parameter affecting the differences between the growth in broth and minced fish mediums. As shown in Table 1, all tested sensory properties of samples containing ZMEO up to 0.5% were acceptable, but additional levels of ZMEO (0.7%) were not desirable for overall acceptance and taste characteristics. However, color, odor, and texture were acceptable in samples containing 0.7% ZMEO. Increasing the concentration of ZMEO up to 0.7% caused bitterness and the strong odor of ZMEO and had a significant negative effect on the overall acceptance scores and taste. Samples containing 0.5% of ZMEO received the highest sensory score for all parameters except color.

**TABLE 1:** Sensory evaluation score in different treatments of minced fish containing 0.3%, 0.5% and 0.7% of ZMEO

Sensory property	Concentration of ZMEO		
	0.3	0.5	0.7
Overall acceptance	4 ± 0.5^a^	4.48 ± 0.8^a^	2.1 ± 0.4^b^
Flavor	3.53 ± 0.4^a^	3.96 ± 0.91^a^	2.46 ± 0.25^b^
Color	3.83 ± 0.91^a^	3.95 ± 0.5^a^	3.83 ± 1.1^a^
Odor	4.06 ± 0.88^a^	4.10 ± 0.81^a^	3.60 ± 0.56^a^
Texture	3.36 ± 0.95^a^	4.03 ± 0.5^a^	3.50 ± 0.98^a^

Note

Mean ± Standard deviation

Values with different letters are significantly different (*p* < .05) in each row (1 = dislike extremely, 2 = dislike moderately, 3 = neither like nor dislike, 4 = like moderately, and 5 = like extremely).

In second step of this study, the fish isolate of *L. monocytogenes* was inoculated in real food. The antilisterial effect of *Zataria multiflora* Boiss. essential oil in fish broth medium and rainbow trout fish medium incubated at three different temperatures of 12, 22, and 37°C for 0, 24 and 48 hr is shown in Table 2. As shown in Table 2, the population increase is relatively similar for 0.01% EO in fish broth and 0.5% EO in minced fish, at the same temperatures of 12 and 22°C. While the population of *L. monocytogenes* remained relatively constant at the inoculation level of 10^7^ cfu/ml (or g) at 12°C in broth and minced fish mediums for 2 days, an increase in its populations was observed for both mediums incubated at 22°C and 37°C. The population of *L. monocytogenes* in samples incubated at 22°C and 37°C increased to 10^11^ cfu/g after 48 hr. Additionally, the higher decrease in the population of *L. monocytgenes* in both mediums containing more concentration of ZMEO was observed indicating the antilisterial effect of ZMEO. However, the increase in temperature significantly decreased the antimicrobial efficiency of ZMEO. For example, it can be observed that for the broth fish medium, the population of *L. monocytgenes* for the samples containing 0.5% of ZMEO incubated at 37°C was almost equal to the samples containing 0.5% ZMEO incubated at 22°C. Similar results have been obtained for *Bacillus Cereus* by exposure to ZMEO, when the temperature was decreased from 30°C to 10°C (Misaghi & Basti, 2007). In the study of Abdollahzadeh et al. (2014), 0.8% and 1.2% of thyme EO, reduced the viable counts of *L. monocytogenes* to below 2 log cfu/g in minced fish at 4°C after 144 hr. The population of *L. monocytogenes* increased at the end of incubation for all treated samples, indicating the reduction of antimicrobial effects of ZMEO during the incubation period. This finding might be due to the stronger antilisterial activity of thyme EO at earlier stages of exposure in terms of the disruption of the cell wall, damage to the organelle, and clumping of cytoplasmic materials, even at low concentrations of EO (Rasooli et al., 2006). The population of *L*. *monocytogenes* in the control group incubated at 37°C was significantly higher than the viable count of control group that remained at room temperature in fish broth. These results were in accordance with the findings of Hadjilouka et al. (2016). Although *L. monocytogenes*, due to its psychotropic properties, can be considered a dangerous pathogenic agent in foods stored at refrigerator temperature, its population did not show the significant increase at abuse refrigeration temperature (at 12°C). This result was inconsistent with the results of Abdollahzadeh et al. (2014) who found that for the control sample stored at low refrigeration temperature, the initial population had not increased after 6 days of storage (Abdollahzadeh et al., 2014). *Listeria monocytogenes* strains can survive and grow at various environmental conditions such as high concentrations of salt and acid and at low temperatures. Although the growth of *L. monocytogenes* at low temperatures is reduced, however, it can survive even at −0.5°C (Buchanan et al., 2017). Studies on the effect of temperature fluctuations by using time/temperature profiles have shown that seasonal temperature variation and temperature abuse during transportation can increase growth probabilities of pathogens. For example, the retail storage temperature and duration can significantly increase *L. monocytogenes* population up to 3.0 log cfu/g (Zeng et al., 2014). *L. monocytogenes* as a psychrotrophic bacteria grow better than nonpsychrotophic bacteria at temperatures ranging from 9 to 16°C. However, narrow temperature changes might not significantly affect lag time and microbial growth (Swinnen et al., 2005).

**TABLE 2:** Inhibitory effect of *Zataria multiflora* Boiss. essential oil on growth of *Listeria monocytogenes* in fish broth medium and rainbow trout fish medium incubated at three different temperatures of 12, 22, and 37°C for 0, 24 and 48 hr

Counts of L. monocytogenes (cfu/ml or cfu/g)									
	12°C			22°C			37°C		
	48 hr	24 hr	0 hr	48 hr	24 hr	0 hr	48 hr	24 hr	0 hr
Fish broth									
0.01%	7.13 ± 0.05^a^	7.02 ± 0.03^a^	7.05 ± 0.08^a^	7.12 ± 0.03^a^	8.06 ± 0.07^b^	8.45 ± 0.03^c^	7.12 ± 0.03^a^	8.04 ± 0.05^b^	8.75 ± 0.23^d^
Control	7.24 ± 0.07^a^	7.11 ± 0.12^a^	7.31 ± 0.11^a^	7.15 ± 0.02^a^	9.33 ± 0.05^b^	9.5 ± 0.01^c^	7.12 ± 0.03^a^	10.05 ± 0.15^d^	10.35 ± 0.04^e^
Fish meat									
0.1%	7.32 ± 0.24^a^	7.12 ± 0.07^a^	7.7 ± 0.05^b^	7.31 ± 0.12^a^	9.01 ± 0.03^c^	9.47 ± 0.03^d^	7.25 ± 0.11^a^	9.08 ± 0.06^c^	9.53 ± 0.04^d^
0.5%	7.11 ± 0.25^a^	7.02 ± 0.06^a^	6.9 ± 0.03^a^	7.06 ± 0.08^a^	8.7 ± 0.03^b^	9.03 ± 0.04^c^	7.14 ± 0.12^a^	8.02 ± 0.06^d^	8.6 ± 0.01^e^
Control	7.02 ± 0.03^a^	7.06 ± 0.04^a^	7.1 ± 0.16^a^	7.02 ± 0.05^a^	10.2 ± 0.22^b^	10.6 ± 0.09^c^	7.05 ± 0.05^a^	10.5 ± 0.21^c^	11.04 ± 0.06^d^

Note

Mean ± Standard deviation.

Mean values with different letters within the same column are significantly different compared to controls (*p* < .05).

Carvacrol as the main ZMEO component has shown synergetic effects with other ZMEO components such as thymol and citral against *L. monocytogenes,* which reduced the combined inhibitory concentration up to 50% (Silva et al., 2015). Carvacrol and thymol as two terpenes with comparable structures have the capacity to penetrate or disrupt lipid structures, affect cytoplasmic membrane, damage its integrity, and change the motive power. They can interfere cell membrane sulfur‐containing biomolecules, resulting the release of lipopolysaccharides in Gram‐positive bacteria. Generally, EOs can affect the membrane as antimicrobial agents by the following mechanisms: (a) increase of permeability, (b) action on intra‐ and extra‐cellular ATP and on ATPases, (c) cytoplasm coagulation, (d) alteration of membrane fatty acids, (e) effect on membrane proteins, (f) leakage of cytoplasmic constituents of metabolites and ions, and (g) denaturation of several enzymes. These mechanisms lead to the reduction in the target bacteria counts by the use of effective concentrations of EOs in foods.

It should be noted that in fish processing plants, the repeated‐presence of same subtypes or persistence of *L. monocytogenes* can be associated with cross‐contamination via raw seafood, seawater, and by attaching to skinning and slicing machines, salting and packaging units (Ferreira et al., 2014). There have been studies reporting resistance of *L. monocytogenes* to disinfectants including phenol, aldehydes, and alcohols as similar components of essential oils. The overexpression of virulence genes and increased minimum inhibitory concentrations (MICs) occur by frequent application of same disinfectants or NaCl below sublethal recommended concentrations (Faleiro et al., 2003; Lundén et al., 2003). Other than the presence of disinfectants at subMICs, exposure to sublethal stress factors may result to adaption of *L. monocytogenes* (Bergholz et al., 2012a). However, adapted strains might not survive at postprocessing stages such as lowering water activity (a_w_) by using salts in food formulations which should be studied (Ferreira et al., 2014). Thus, the possible synergistic effect of preservatives such as essential oils and salts as an aspect of hurdle technology requires further considerations.

## 4 CONCLUSION

In case of the use of EOs and sodium chloride in sublethal concentrations, hurdle techniques should be used to reduce their effects on sensory properties and hypertension, respectively. Application of NaCl and acid resulted in different growth patterns of *L. monocytogenes* strains. This microorganism can tolerate low temperatures and high contents of acid and salt. It is important to evaluate the adaption of *L. monocytogenes* in food formulations and processing conditions such as different temperatures, acidification and salting. Further, antimicrobial strategies such as use of natural preservatives such as phytochemicals is promising. Although in our study both the food and clinical strains of *L. monocytogenes* were tolerant toward different concentrations of acid and salt; however, the overall stress exposure in foods may alter the growth patterns. The growth of *L. monocytogenes* isolated from rainbow trout fish during its exposure to ZMEO was studied in two rainbow trout fish mediums at different temperatures for the first time. Our results showed that ZMEO in safe and sensory acceptable concentrations cannot inhibit the growth of *L. monocytogenes* at abuse, room, and optimum temperatures and should be coupled with other preservatives. However, other than investigating the effect of natural preservatives on the growth of pathogenic bacteria, their effect on the potential virulence properties of target microorganisms through molecular methods should be studied. It should also be considered that higher concentrations of EOs and sodium chloride at lower pH values is needed in vivo compared to in vitro conditions as various intrinsic and extrinsic properties influence EOs antimicrobial activities in a food matrix.
